# Pulmonary tumor with Notochordal differentiation: a case report and morphologic, Immunohistochemical and molecular study of benign Notochordal cell tumor originating in the lung

**DOI:** 10.1186/s13000-021-01157-5

**Published:** 2021-10-30

**Authors:** Kai Song, Xiaojing Ma, Jinghong Xu, Lirong Chen

**Affiliations:** Department of Pathology, The Second Affiliated Hospital Zhejiang University School of Medicine, Hangzhou, China

**Keywords:** Benign notochordal cell tumor, Lung, Molecular, EGFR, Case report

## Abstract

**Background:**

Extraosseous benign notochordal cell tumor is extremely rare, and there are only five reported cases worldwide. The presented case of pulmonary primary benign notochordal cell tumor is the sixth case, but the first to report the deletion mutation of EGFR gene exon 19.

**Case presentation:**

The patient was a 50-year-old asymptomatic woman, who had been followed up for 3 years for a nodule in the right lung. After ten months of the wedge resection, the patient is alive without evidence of recurrence or metastasis. The tumor was 7 mm in diameter and was well demarcated. The tumor was consisted of a sheet of large round vacuolated cells with small and bland nuclei. No connective tissue containing blood vessels or inflammatory cell infiltration was detected in the ﻿stroma. The tumor was positive for CK AE1/AE3, Vimentin, S100 and Brachyury. EGFR gene mutation and amplification were not detected.

**Conclusions:**

We firstly reported the positive immunohistochemical staining for EGFR and the negative molecular results of EGFR gene of pulmonary primary benign notochordal cell tumor. Due to the rarity of this tumor, more reports are needed to explore pathological characteristics, especially the molecular characteristics, in order to better understand the nature of tumors.

## Background

Benign notochordal cell tumors (BNCTs) are tumors that originate from notochordal remnants [1], and was firstly reported by Yamaguchi et al. [2] in 2002. Compared with chordoma, BNCT is a relatively common intraosseous lesion, which can be seen in nearly 20% of adult autopsies according to a Japanese investigation [3, 4]. The distribution of BNCT is similar to chordomas, axial skeleton, especially the lumbo-sacral region and clivus are the most common sites. Extraosseous chordomas have been reported in soft tissue and lung [5–7], while extraosseous BNCTs were only occurred in lung [3, 8–10], and there were only five reported cases. The pulmonary primary BNCT was first reported by Kikuchi [3] in 2011.

Here, we report the sixth case of extraosseous BNCT originating in the lung, which was confirmed by pathological, immunohistochemical and molecular findings, and review the associated reported literatures to summarize the clinical and pathological features of this rare tumor of the lung.

## Case presentation

### Clinical findings

**Fig. 1:**
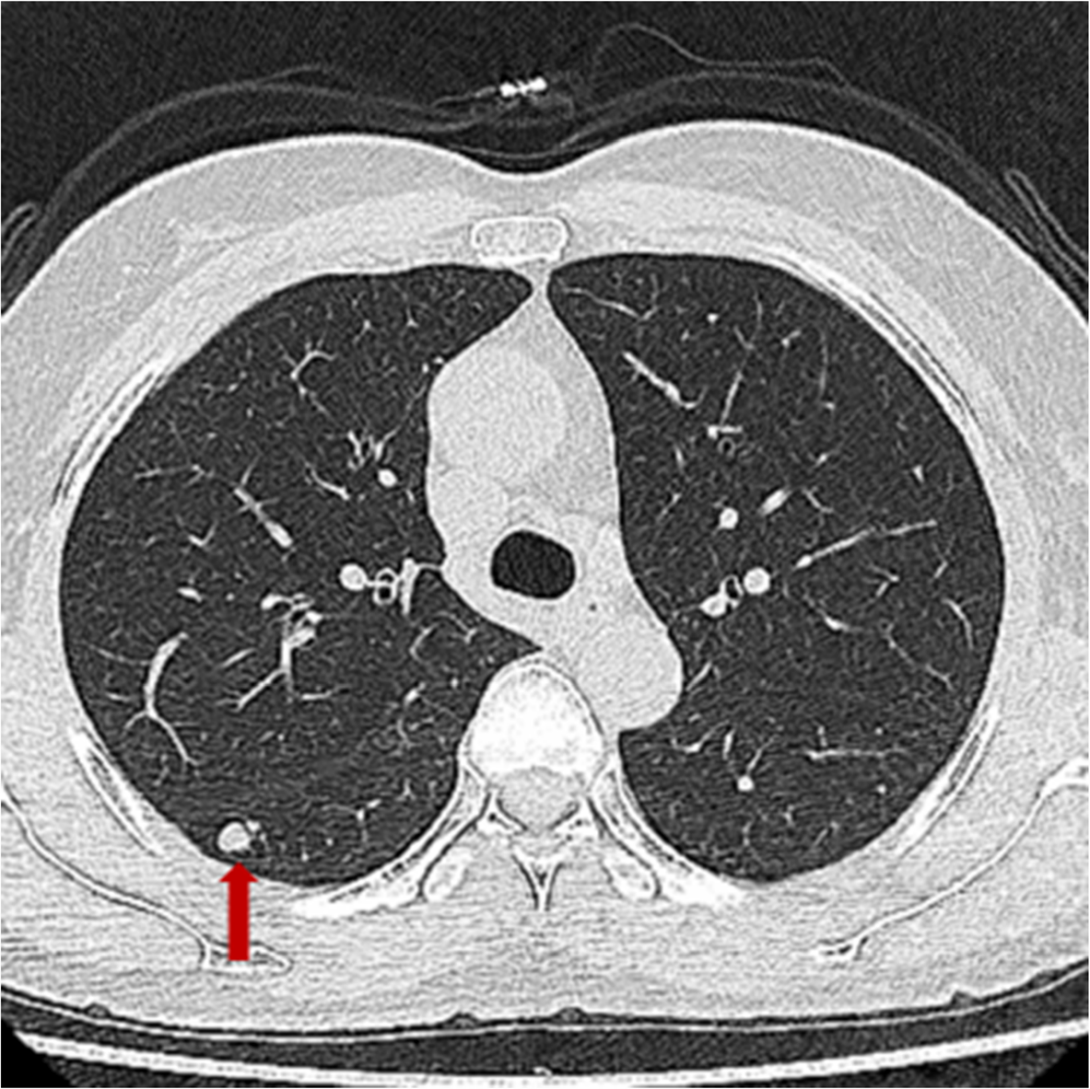
Chest computed tomographic image reveals a well-demarcated, round, solid nodule in the right lung (red arrow)

The patient was a 50-year-old asymptomatic Chinese woman, who had been followed up for a lung nodule in the past 3 years and was not taking any medications. Chest computed tomography (CT) demonstrated a small round tumor with clear margin, which was 7 mm in diameter, in segment 8 of the right lung (Fig. 1), and was diagnosed as a benign lesion. The whole body bone scintigraphy did not detect any bone lesion. The levels of serum tumor markers, including alpha fetal protein (AFP), carcino-embryonic antigen (CEA), β-human chorionic gonadotropin (β-hCG), neuron-specific enolase (NSE), carbohydrate antigen199 (CA199), squamous cell carcinoma antigen, cytokeratin-19-fragment CYFRA21-1 (CA211), cancer antigen 242 (CA242), cancer antigen 153 (CA153) and cancer antigen 125 (CA125) were not elevated. A wedge resection was conducted. Neither pleural adhesion, tumor dissemination, nor an accumulation of pleural effusion was noted during the operation. After ten months post surgery, there is no adverse or unanticipated event happened, and the patient is alive without evidence of recurrence or metastasis.

### Pathological findings

**Fig. 2:**
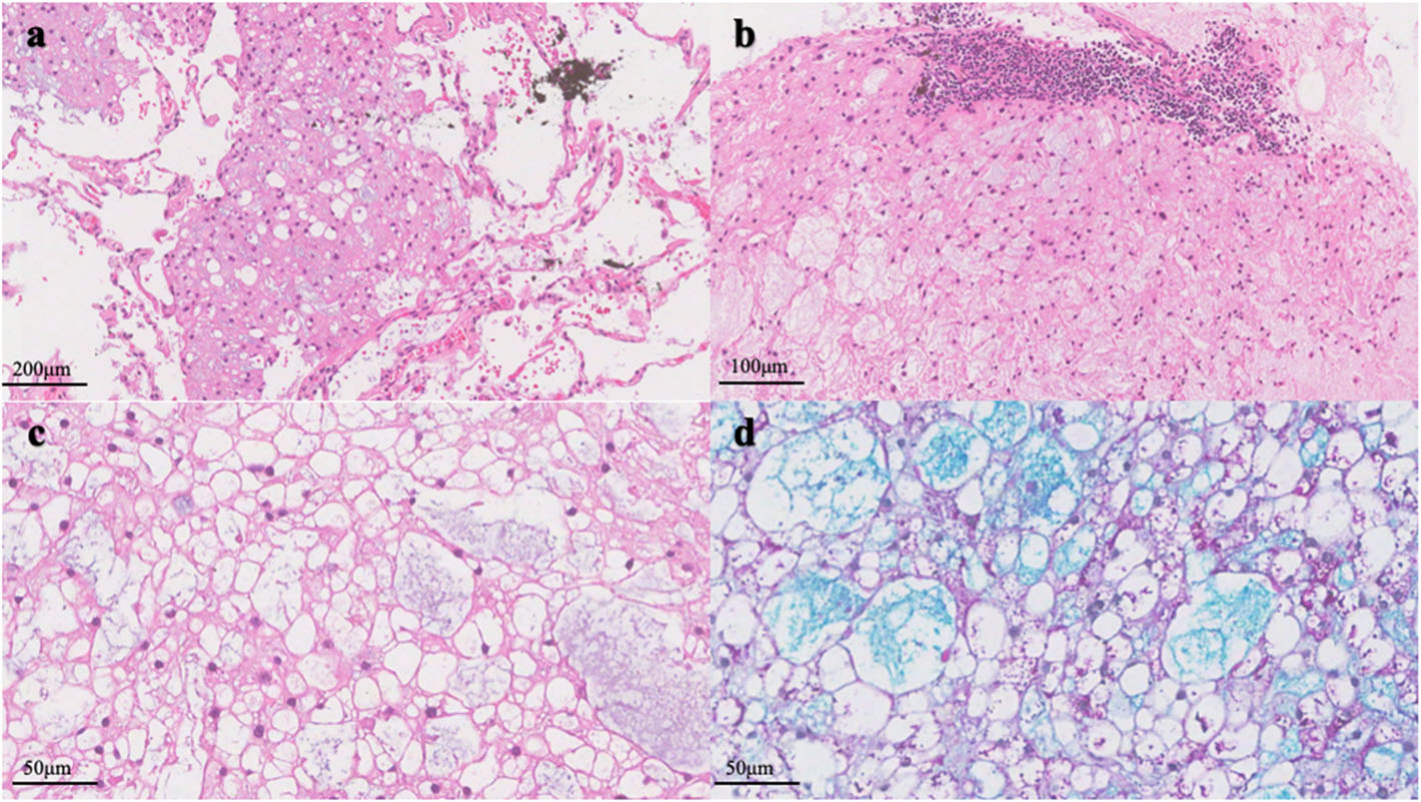
The tumor was unencapsulated, but well demarcated, minimal destruction of the alveolar structure (a) and cuffs of lymphocytes were observed at the board (b). The tumor cells were vacuolated and resembled mature adipose cells with bland nuclei (c), intracellular mucous (d) was positive for AB/PAS staining

**Fig. 3:**
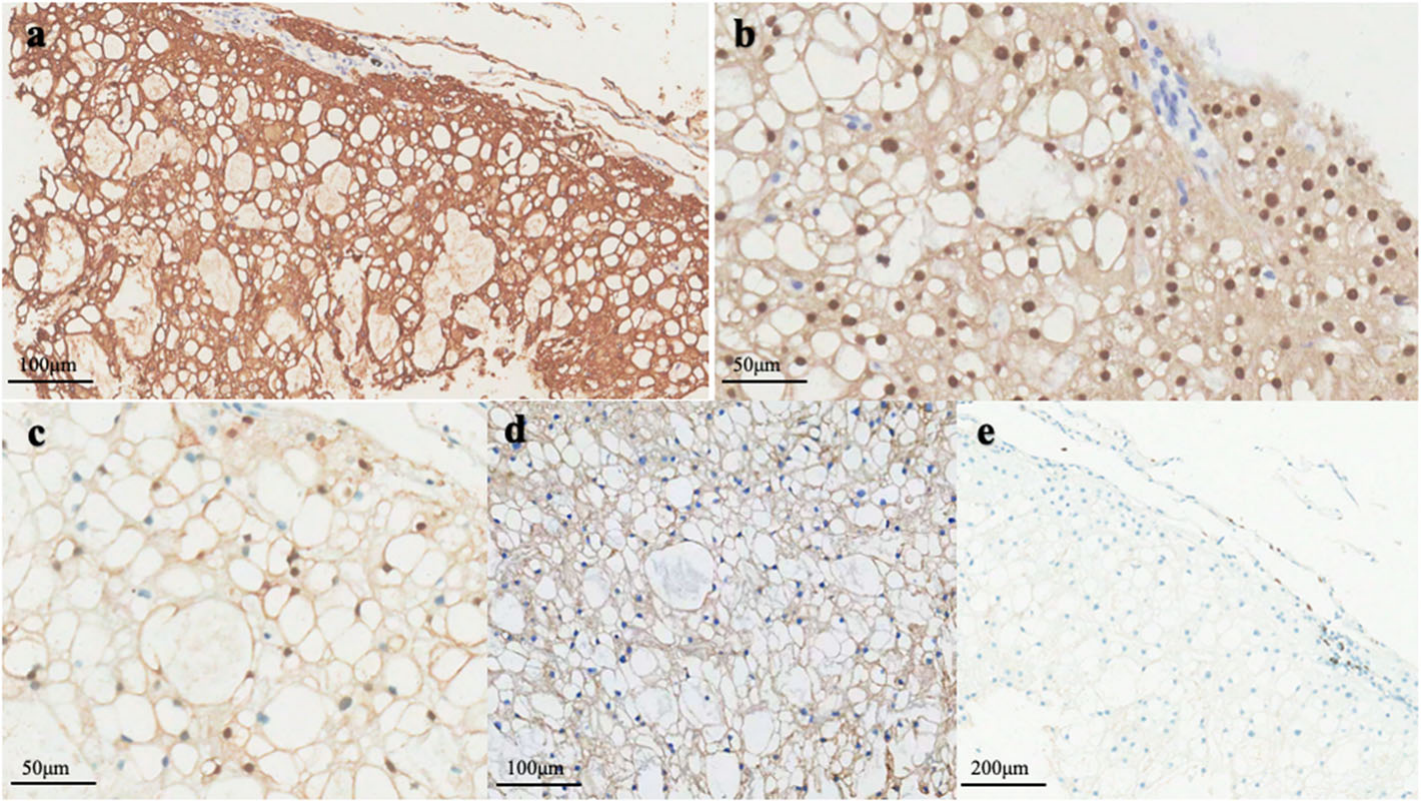
The tumor cells were positively immunohistochemical staining for CK (AE1/AE3) (a), Brachyury (b), S100 (c), EGFR (d) negatively staining for TTF-1 (e)

**Fig. 4:**
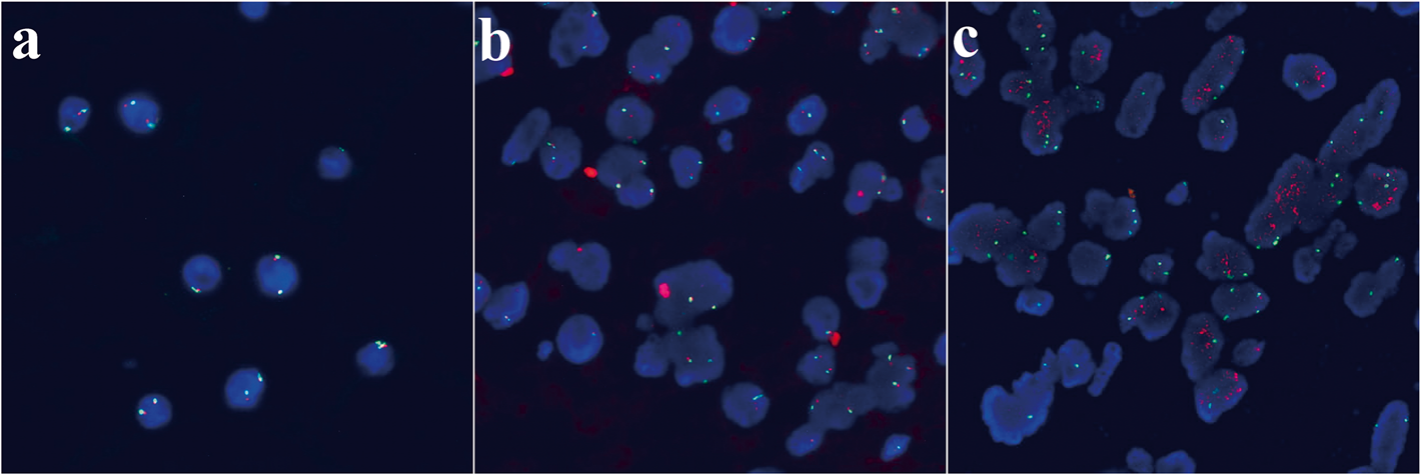
The fluorescence in situ hybridization study showed no evidence of the EGFR gene amplification (a). Negative control (b) and positive control (c) were set

Macroscopically, an unencapsulated, but well demarcated tumor, measuring 7 mm in diameter, was seen adjacent to visceral pleura. On the sectioning, the tumor was white-gray and jelly-like, with a microcystic change. A small amount of mucus-like fluid came out. Histologically, the tumor was consisted of a sheet of large round cells with microcystic appearance, due to the uni-, multi- and less vacuolated cells. The uni-vacuolated cells resembled mature adipose cells and the multi-vacuolated cells resembled physaliphorous cells of chordoma. Most nuclei were small and round, and placed peripherally, but few of them were larger and in an irregular shape. Apparent nucleoli, atypia or mitosis was not encountered. Myxoid substance was observed both in the cytoplasm of vacuolated cells and microcystic space, which was positively staining for alcian-blue/periodic acid-Schiff staining (AB/PAS), and ﻿extracellular matrix was not identified. Neither connective tissue containing blood vessels nor inflammatory cell infiltration was detected in the stroma. Cuffs of lymphocytes were observed at the board. Minimal destruction of the alveolar structure was showed at the periphery of the tumor (Fig. 2). Immunohistochemically, the tumor was positive for immunohistochemical markers for CK AE1/AE3, Vimentin, S100, Brachyury, epithelial membrane antigen (EMA) and epidermal growth factor receptor (EGFR). The ratio of Ki-67-positive cells within the tumor was extremely low (1%). Thyroid transcription factor-1 (TTF-1), P63, P53 and GATA-3 were negatively staining (Fig. 3). Molecularly, amplification and mutation of EGFR gene were not detected by fluorescence in situ hybridization (FISH) analysis (Fig. 4) and the amplification refractory mutation system (ARMS-PCR), respectively.

## Discussion and conclusions

Pulmonary BNCT is extremely rare, and the incidence is still unknown [4, 10]. To our best knowledge, there are only five cases which have been reported so far, in the English literature via PubMed search (Table 1). The presented study is the sixth case. Of these six cases (Table 1), female patients are twice as much as male patients, and the age range was 38 to 57 years old (median, 49 years old). All the patients came from Asian countries, four patients were Japanese, and the other two came from China. Five patients were detected with nodules in single lobe of the lung (four cases in the right lung and one case in the left lung), and only one case presented as bilateral nodules. Six out of seven nodules were adjacent to the visceral pleura, while one nodule was revealed within the lung parenchyma. Previously reported patients remained alive and asymptomatic with no evidence of recurrence or metastasis after 1-5 years since the operation. (Table 1).

**Table 1:** Clinical Features of the six reported lung BNCTs

Case No.	Reference	Area	Age (y)	Sex	Tumor		Diameter (mm)	Follow-up
					Side	Location		
1	Kikuchi Y [3]	Japan	48	M	Right	Within the parenchyma	15	1 year
2	Kikuchi Y [3]	Japan	38	F	Right	Beneath the visceral pleura	15	1 year
3	FY, Lee [9]	China	53	F	Bilateral	Beneath the visceral pleura	10, each	2 year
4	Y Takahashi [10]	Japan	57	F	Right	Adjacent to the ﻿visceral pleura	10	5 year
5	M Shintaku [8]	Japan	41	M	Left	﻿Subpleural	12	20 months
6	Presented	China	50	F	Right	Adjacent to the ﻿visceral pleura	7	10 months

Macroscopically, the tumors were well demarcated, with a tumor size ranging from 7 to 15 mm (median, 10 mm) in diameter. On the sectioning, the tumors were gray-white and showed central cystic changes, except the report in Y Takahashi et al. [10]. Microscopically, the previously reported cases and our presented case shared almost identical characteristics. The cells were vacuolated with peripherally located nuclei, mimicking mature adipocytes. The nuclei were small and bland, with no atypia or mitosis, implying the benign nature of the tumors. Furthermore, our case and Y. Takahashi et al. [10]’ s demonstrated a cluster of lymphocytes at the board of the tumor.

Intraosseous BNCTs are characterized by unencapsulated sheets of adipocyte-like vacuolated and less vacuolated cells. They exhibit bland round nuclei and eosinophilic cytoplasm. No mitotic figures are recognized. The tumors lack any intercellular myxoid matrix [2, 4]. These pathological features are identical to those lung BNCTs, including this presented case.

Pulmonary chordoma is extremely rare, to our knowledge, there were only three reported cases [6, 7, 11]. The pathogene sis is unclear, one possible mechanism may derive from multipotent cells in the lung parenchyma or a notochordal remnant with aberrant migration from midline [6]. Because of the aggressive behavior of chordoma, it is important to differentiate BNCT from chordoma. Lobule structure containing cords or nests of atypia notochordal cells and extracellular myxoid matrix are the important histological features of chordoma, while the BNCT lacks such structures. Intraosseous BNCT has recently been recognized to be a potential precursor of classic chordoma [2, 12], while it has not been documented that chordomas arise from BNCT in the lung, pulmonary BNCT is considered a potential precursor of classic chordoma [3, 9, 11].

Besides the chordoma, metastatic tumor should be ruled out first. The whole-body CT, bone scintigraphy, and (18) F-fluorodeoxyglucose-positron emission tomography would be helpful. Other primary pulmonary neoplasms with myxoid background and secondary clear cell tumors, such as myoepithelioma, hamartoma, pleomorphic adenoma, perivascular epithelioid cell tumor, pulmonary myxoid sarcoma with EWSR1-CREB1 fusion should be considered in the differential diagnosis [3, 8–10]. But these were easily ruled out on the basis of pathological and clinical findings and the immunohistochemistry.

Immunohistochemical staining is often used to confirm the diagnosis of BNCT. Typically, BNCT constantly express brachyury, a key transcription factor involved in the early stage of posterior mesoderm development of hemangioblasts and notochord [3, 5, 13, 14]. In the previous literatures, there two different brachyury-positive pattern. Four nodules showed strong immunoreactive for Brachyury, only one nodule was focally positive [10]. In their study, Takahashi et al. analyzed the differences in Brachyury-positive expression between BNCTs and chordomas. They found the ratio was dramatically lower than chordomas [10]. According to Shen et al. [15], those brachyury negative parts might be bound up with the fetal notochordal cells, as they reported that brachyury staining was negative in the BNCT component around chordomas and was negative in fetal notochordal cell rests, which are histopathologically similar to BNCTs. Jambhekar et al. [16] have reported the expression rate of brachyury in chordomas was 90.2%, therefore, some other sensitive but less specific markers, including CK (AE1/AE3), EMA, S100, were usually recommended to be used in combination [9]. In our case, the brachyury-positive pattern was consistent with most of the literatures aforementioned, and CK (AE1/AE3), EMA, S100 were positively stained, strongly suggested notochordal differentiation. Reported skull BNCT typically had a Ki-67 index range from <1 to 3% [17–19], while the lung BNCTs was 1 to 5.2% [3, 8–10], which was even higher. However, the follow-up results showed that no recurrence occurred in patients with a higher Ki-67 index, indicating the benign biological behavior of this tumor [19].

To the best of our knowledge, none of the previous cases of lung BNCT mentioned the molecular features, and we firstly reported the EGFR gene changes. Inspired by the serendipitous positive result of EGFR immunohistochemical staining, EGFR amplification and mutation tests were conducted, and the results were both negative. However, Du reported the EGFR amplification rate in skull base BNCT was 30.8%, which was much lower than that in chordoma [19]. While EGFR gene mutation was not mentioned, which was common in lung adenocarcinomas.

Because of the rarity of the lung BNCT, there is no consensus on treatment at present. Though the existing histological and immunohistochemical features indicate the benign nature of the tumor to some extent, surgical resection and close follow-up seem to be routine treatments. Therefore, more reports are need to explore pathological characteristics, especially the molecular characteristics, in order to better understand the nature of tumors.

## Data Availability

The data sets used and/or analyzed during the current study are available from the corresponding author on reasonable request.

## Abbreviations

BNCT: Benign notochordal cell tumor
EGFR: Epidermal growth factor receptor
FISH: Fluorescence in situ hybridization

## References

[1] Heaton JM, Turner DR (1985). Reflections on notochordal differentiation arising from a study of chordomas. Histopathology..

[2] Yamaguchi T, Yamato M, Saotome K (2002). First histologically confirmed case of a classic chordoma arising in a precursor benign notochordal lesion: differential diagnosis of benign and malignant notochordal lesions. Skelet Radiol.

[3] Kikuchi Y, Yamaguchi T, Kishi H, Azuhata K, Kimizuka G, Hiroshima K (2011). Pulmonary tumor with notochordal differentiation: report of 2 cases suggestive of benign notochordal cell tumor of extraosseous origin. Am J Surg Pathol.

[4] Yamaguchi T, Suzuki S, Ishiiwa H, Ueda Y (2004). Intraosseous benign notochordal cell tumours: overlooked precursors of classic chordomas?. Histopathology..

[5] Tirabosco R, Mangham DC, Rosenberg AE, Vujovic S, Bousdras K, Pizzolitto S (2008). Brachyury expression in extra-axial skeletal and soft tissue chordomas: a marker that distinguishes chordoma from mixed tumor/myoepithelioma/parachordoma in soft tissue. Am J Surg Pathol.

[6] Strano S, Ouafi L, Baud M, Alifano M (2010). Primary Chordoma of the Lung. Ann Thorac Surg [Internet] Elsevier Inc.

[7] Park SY, Kim SR, Choe YH, Lee KY, Park SJ, Lee HB (2009). Extra-axial chordoma presenting as a lung mass. Respiration..

[8] Shintaku M, Kikuchi R (2020). Benign notochordal cell tumor of the lung: report of a case. Pathol Int.

[9] Lee FY, Wen MC, Wang J (2013). Extraosseous benign notochordal cell tumor presenting as bilateral pulmonary nodules. Hum Pathol [Internet] Elsevier Inc.

[10] Takahashi Y, Motoi T, Harada M, Fukuda Y, Hishima T, Horio H (2015). Extraosseous benign notochordal cell tumor originating in the lung: A case report. Med (United States).

[11] Ohya M, Yoshida K, Shimojo H, Shiina T (2018). Multiple primary chordomas of the lung. Respir Med Case Rep [internet] Elsevier.

[12] Yamaguchi T, Watanabe-Ishiiwa H, Suzuki S, Igarashi Y, Ueda Y (2005). Incipient chordoma: a report of two cases of early-stage chordoma arising from benign notochordal cell tumors. Mod Pathol.

[13] Vujovic S, Henderson S, Presneau N, Odell E, Jacques TS, Tirabosco R (2006). Brachyury, a crucial regulator of notochordal development, is a novel biomarker for chordomas. J Pathol.

[14] Sangoi AR, Dulai MS, Beck AH, Brat DJ, Vogel H (2009). Distinguishing chordoid meningiomas from their histologic mimics: an immunohistochemical evaluation. Am J Surg Pathol.

[15] Shen J, Shi Q, Lu J, Wang DL, Zou TM, Yang HL (2013). Histological study of chordoma origin from fetal notochordal cell rests. Spine (Phila Pa 1976).

[16] Jambhekar NA, Rekhi B, Thorat K, Dikshit R, Agrawal M, Puri A (2010). Revisiting chordoma with brachyury, a “new age” marker: analysis of a validation study on 51 cases. Arch Pathol Lab Med.

[17] Lagman C, Varshneya K, Sarmiento JM, Turtz AR, Chitale RV (2016). Proposed diagnostic criteria, classification Schema, and review of literature of notochord-derived Ecchordosis Physaliphora. Cureus..

[18] AlOtaibi F, Guiot M-C, Muanza T, Maio S (2014). Giant Petroclival primary Intradural Chordoma: case report and systematic review of the literature. J Neurol Surg Reports.

[19] Du J, Xu L, Cui Y, Liu Z, Su Y, Li G (2019). Benign notochordal cell tumour: Clinicopathology and molecular profiling of 13 cases. J Clin Pathol.

